# Trust and the ethical challenges in the use of whole genome sequencing for tuberculosis surveillance: a qualitative study of stakeholder perspectives

**DOI:** 10.1186/s12910-019-0380-z

**Published:** 2019-07-04

**Authors:** Carly Jackson, Jennifer L. Gardy, Hedieh C. Shadiloo, Diego S. Silva

**Affiliations:** 1 Faculty of Health Sciences, Simon Fraser University, BLU 11300 – 8888 University Drive, Burnaby, BC V5A 1S6 Canada; 2 British Columbia Centre for Disease Control, Vancouver, BC Canada; 3 School of Population and Public Health, the University of British Columbia, Vancouver, BC Canada; 4 Sydney Health Ethics, Faculty of Medicine and Health, University of Sydney, Sydney, Australia

**Keywords:** Trust, Surveillance, Tuberculosis, Whole genome sequencing, Qualitative, Empirical ethics

## Abstract

**Background:**

Emerging genomic technologies promise more efficient infectious disease control. Whole genome sequencing (WGS) is increasingly being used in tuberculosis (TB) diagnosis, surveillance, and epidemiology. However, while the use of WGS by public health agencies may raise ethical, legal, and socio-political concerns, these challenges are poorly understood.

**Method:**

Between November 2017 and April 2018, we conducted semi-structured interviews with 22 key stakeholders across the fields of governance and policy, public health, and laboratory sciences representing the major jurisdictions currently using WGS in national TB programs. Thematic analysis of the interviews was conducted using *NVivo 11*.

**Results:**

Respondents identified several ethical and practical challenges associated with WGS in TB care and surveillance, all related to issues of trust, including: 1) the power of public health; 2) data sharing and profits derived from surveillance efforts; and 3) concerns regarding who has access to, and can benefit from, the technology. Additional challenges included: the potential utility that WGS adds to a public health program, the risks associated with linking necessary epidemiological metadata to the genomic data, and challenges associated with jurisdictional capacity to implement the technology.

**Conclusions:**

Successful implementation of WGS is dependent on fostering relationships of trust between those working with genomics technology and those directly impacted by it, including clinicians. Building trust (a) between the public and the public health agencies and (b) within public health agencies themselves is critical due to the inherent complexity of WGS and its implementation for communicable disease control purposes.

**Electronic supplementary material:**

The online version of this article (10.1186/s12910-019-0380-z) contains supplementary material, which is available to authorized users.

## Background

Whole genome sequencing (WGS) has dramatically changed public health microbiology, allowing for rapid diagnosis of infections, antibiotic resistance prediction, and accurate outbreak identification and reconstruction [1], while positioning us for a future in which individualized medicine for specific infections is possible [2]. As WGS becomes an increasingly important tool in public health, its successful implementation depends on complex human and systems factors beyond simply laboratory and clinical practice. Critical examination of these challenges is necessary in order to fully realize these benefits. In part, understanding the ethical challenges associated with the use of WGS is vital because public health is not merely a scientific endeavour, but one built on the normative ideal of social justice [3].

In order to successfully implement WGS into pre-existing health systems, trust must be fostered between relevant stakeholders. Trust, as used in the ethics literature, may refer to several different concepts: public trust (e.g., trust of public bodies like government), interpersonal trust (i.e., trust between persons or groups), and institutional trust (i.e., trust in the pre-existing structures of sociopolitical and economic organizations) [4]. Trust may be a perception or attitude one holds regarding another actor’s trustworthiness; ‘trustworthiness’ is the honesty and integrity of the actor’s actual behaviour [5]. Further, trust suggests that one party is vulnerable and dependent upon others to act in the right manner, including taking the trustee’s interest into account [5]. This, in turn, suggests that there may exist a power imbalance between the person or group *to be trusted* (i.e., those in positions of power) and the person or group *who is trusting* (i.e., those in positions of disempowerment). In both research and policy, we ought to be aware of the reasons that power imbalances exist in particular contexts, including histories of colonialism and other sociopolitical factors that lead to the marginalization of certain groups of people.

The broader public health and bioethics literature related to issues of trust posits that the values that underpin the concept of trust are necessary for the functioning of health systems and, in turn, health promotion in individuals [6]. Issues of trust related to public health—for example, the power that the enterprise of public health holds in justifying public health actions [7], the fear of exploitation with regard to intellectual property for financial gain in the context of data-sharing [7, 8], and ensuring equitable access to new technologies [9]—have been explored in the literature and identified as a key factor to advancing and implementing new genetic diagnostics for personalized medical care and disease control [7, 10, 11]. In this paper, we used the idea of trust to denote the disposition to trust and being trustworthy in a descriptive rather than a normative or proscriptive sense; it is used as a concept to help organize what we will argue are the main themes from our interviews.

Despite the depth of literature on WGS and trust in the human genomics context at the clinical or bedside level, there is very little written regarding the use of WGS in public health microbiology, including the ethical challenges associated with WGS in public health, the context surrounding these challenges, what can be done to mitigate or resolve these challenges, and what role trust may play in the implementation of public health genomics. For example, genomics is being increasingly used in tuberculosis (TB) diagnosis, surveillance, and epidemiology, with many regional and national public health agencies in low-incidence settings - which are often times high-income countries (HIC) - routinely sequencing all culture-positive TB isolates. Traditionally, TB has been characterized as a “disease of poverty”, meaning persons of lower socioeconomic status are more susceptible to contracting the infection and developing the disease [6]. Therefore, using WGS for TB diagnosis, surveillance, and outbreak investigation raises multiple ethical challenges, including questions around privacy and confidentiality [6, 7], who has access to information generated via WGS and how this information can be used [8], and questions of stewardship regarding the creation and safekeeping of data [8, 12], all of which are heightened due to the socioeconomic marginalization of those whom it most affects.

To our knowledge, this project is the first of its kind to explore the ethical and related legal and socio-political concerns associated with the use of WGS by TB laboratories and programs by interviewing key stakeholders directly impacted by this technology. We explore these concerns through a qualitative analysis of the perspectives of government officials, policymakers, and those working directly in TB care and prevention. We note at the outset that this analysis is meant to be a descriptive analysis of these concerns as elucidated by the perspectives of these key stakeholder groups, rather than a proscriptive commentary. The results we present here are exploratory in nature and contribute to the existing literature detailing ethical concerns associated with the broader use of genomics for infectious disease control and the influence trust holds in the successful implementation of genomic technologies and next-generation sequencing in public health.

## Methods

### Sampling and recruitment

We obtained ethics approval from Simon Fraser University (No. 2017 s0485). We recruited participants through purposive sampling – compiling a list of prospective participants through our existing network of domain experts and targeted searches for individuals working with WGS for TB care and surveillance – and snowball sampling [13], whereby participants identified other potential recruits. Interviews were conducted until saturation was achieved, i.e., when no new ideas arose from the data [13]. In total, we conducted 22 interviews. Participants’ professional fields included governance and policy (including persons with a governance role within public health organizations charged with TB control), public health, and laboratory scientists working with genomics. Many participants had overlapping professional duties. Participants represented those jurisdictions currently using WGS for TB, including Canada, the United States, and the United Kingdom, as well as international organizations representing global and low- and middle-income (LMIC) perspectives. All participants provided written informed consent.

### Data collection

Interviews were administered by phone and digitally recorded between November 2017 and April 2018. Each interview lasted approximately 60 min and all were conducted by the same team member (DSS) using a semi-structured interview guide with open-ended questions; we opted for this approach in this exploratory study as it allows participants to provide more detail as they see fit and as guided by the interviewer [14]. The research team developed the guide after reviewing the existing literature regarding ethical challenges in the implementation of new genetic technologies primarily with regards to infectious disease surveillance and public health. The interview guide was also informed by the global health governance literature and previous experience of the research team in conducting interviews on similar themes. (See Additional file 1 for a summary of questions included in the interview guide). The guide was iteratively refined over the course of the interviews as the team learned more about the topic from the participants, allowing the research team to better understand emerging concepts repeatedly raised by interviewees. This interview guide was relied on more heavily in the beginning of the interview to prompt discussions with participants. As the interview progressed, conversation flowed more naturally between the participant and the interviewer (DSS) as logical follow-up questions were pursued following the information offered by the participant. In this way, as data collection progressed, the interview guide was used more like a checklist to ensure all topics had been addressed in the interviews.

### Analysis

We used thematic analysis to analyze the data [14]. The digital recordings of each interview were sent to a professional transcription company and transcribed *verbatim*. One member of the research team (CJ) verified the accuracy of each transcript. Transcripts were then line-by-line coded using *Nvivo 11* by the same team member using a codebook developed by two members of the research team (CJ and DSS), who first independently coded three interviews representing a range of professional perspectives. These initial codes were compared for overlap between CJ and DSS, and the initial codebook was built; it was further refined during the analysis, with codes added or collapsed as needed.

Two team members not participating in the line-by-line coding (DSS and JG) prepared analytic memos of each transcript describing the high-level themes in each interview. Together with the line-by-line coding, researcher reflexivity was facilitated through systematically constructing the themes together across multiple readings of the transcripts and in two full-day face-to-face meetings. This approach ensured that our analysis was comprehensive and allowed the team to develop a deeper understanding of key themes.

**Fig. 1:**
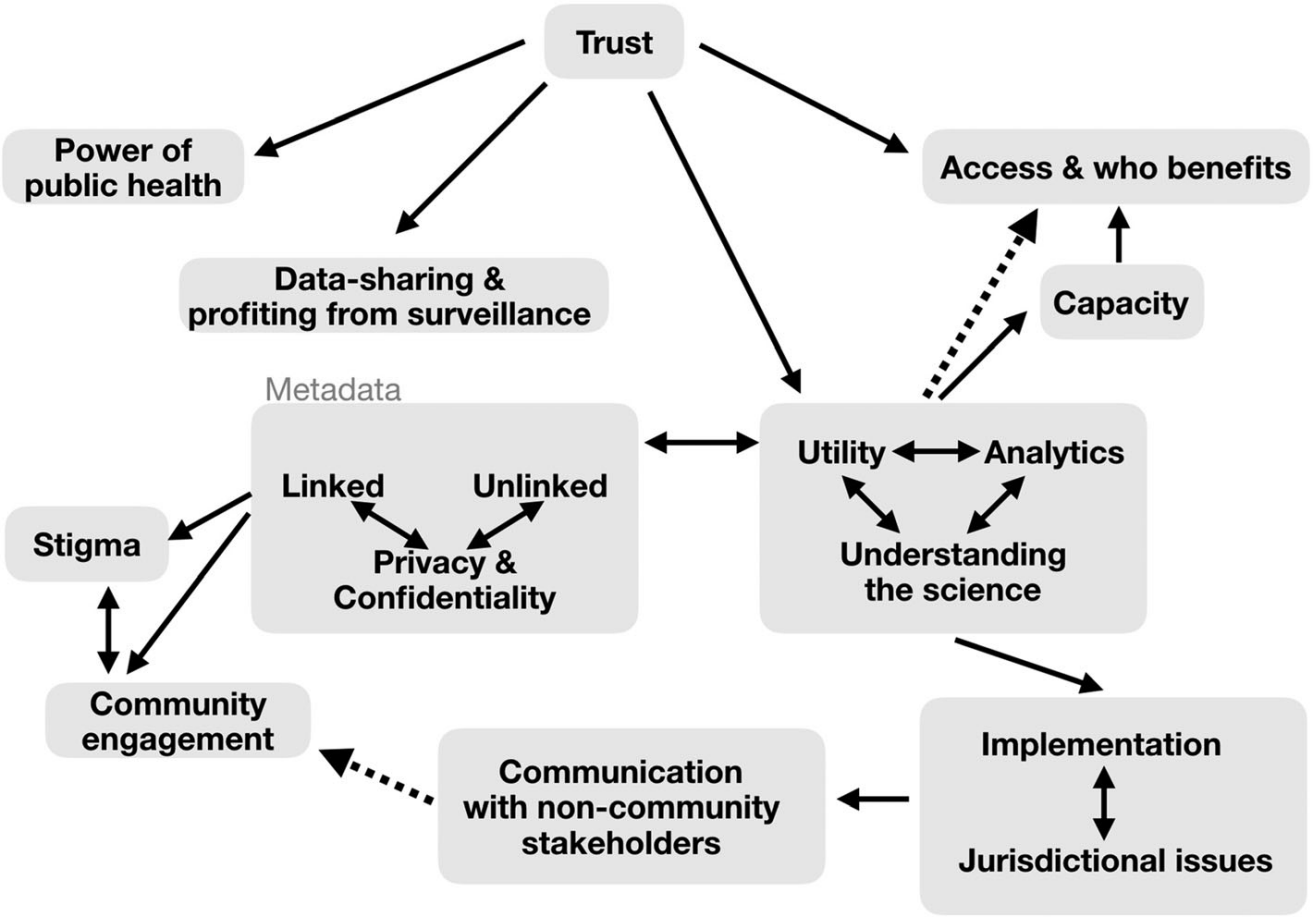
Map of relationships between key concerns in relation to trust. → concept flows from previous concept. ↔ concepts have endogenous relationship, influencing each other.⇢concept has indirect relationship with connecting concept

Once we had determined the overarching themes from the data, the team jointly created Fig. 1 and Table 2, which were shared with the participants in a process sometimes referred to as a ‘member’s check’. Nine participants provided feedback on the team’s assessment of the main themes, which led to further refinement of the themes and the language used in the Results section below.

## Results

Our 22 participants worked primarily in governance/policy and public health, and predominantly in North America, though we did also have participants working in laboratories, as well as international organizations (Table 1).

**Table 1:** Participant Demographics

Regional Representation	Canada	United States	United Kingdom	International Organizations	Totals
Governance and Policy^a^	5	2	0	1	8
Public Health	4	3	1	0	8
Laboratory	1	1	1	3	6
Totals:	10	6	2	4	–

^a^ Includes government officials and individuals in governance roles within public health institutions

Participants identified several key ethical challenges associated with the use of WGS for TB care and surveillance. We have developed a model of the relationships between the key findings (Fig. 1), as well as definitions and illustrative quotes that expand upon the concepts in the model (Table 2). The remainder of this section will explain and contextualize this figure and table. In addition to the table, direct verbatim quotes are provided throughout the results section to further support the ethical challenges highlighted by participants. Throughout this section, we refer to participants by their primary job descriptors (i.e., governance officials, public health workers, or laboratory staff) to protect their anonymity. Any quotes attributed to a participant are identified using their primary job descriptor and participant number. The results did not differ between participants on the basis of geography.

**Table 2:** Summary table of terms used in Fig. 1 with illustrative participant quotes

			Item	Definition/ Operationalized Description	Illustrative Quote
		1	Trust	Trust is an perception or attitude one holds regarding another actor’s trustworthiness in their behaviour; here ‘trustworthiness’ being the honesty and integrity of the actor’s actual behaviour. This trust may be built on relationships in which both parties have proven their trustworthiness or, in other cases, may be blind trust in those with the power over the data. Regardless, trust suggest that one party is vulnerable and dependent upon others to act in the right manner according to the particular situation, including taking the trustee’s interest into account (McLeod, 2015). In the context of WGS for TB surveillance, issues around trust (or lack thereof) may be present in many relationships including, but not limited to: 1) between health care workers and laboratory staff, 2) the public and the medical community and 3) for concerns of data protection for both privacy and confidentiality purposes, as well as academic.	*Well, I think, ultimately, that is what happens, right, is that sort of trust is like – if the folks who are the expert in the technology are saying that these are people who are likely related or that this is like – these are definitely a cluster, then I think you do kind of have to take that on at face value. (Interview #14, Governance). It takes ages to build [trust], and if you make a misstep, the whole thing could be gone. So you have to just be very careful, very open, never to make it seem like you’re on the make. Just be a – and actually, it’s not that hard. You just be a, you know, decent, honest person, and keep people in the loop. (Interview #16, Lab)*
Issues of trust in relation to new technologies previously discussed in the literature		2	Data sharing and profitting from surveillance	Fear of the potential that profits may be derived from data acquired through surveillance activities. In conjunction with data sharing concerns previously detailed in the literature, this encompasses the fear that if data is shared with international surveillance databases, the data can then be used to developed new diagnostics and/or drugs that will then be sold back to countries at high, potentially unaffordable, prices. Fear of the potential that profits may be derived from data acquired through surveillance activities. In conjunction with data sharing concerns previously detailed in the literature, this encompasses the fear that if data is shared with international surveillance databases, the data can then be used to developed new diagnostics and/or drugs that will then be sold back to countries at high, potentially unaffordable, prices.	*They would – they’re pretty hesitant and what has been done in the past is that if they have volunteered to provide data that companies can come in, identify potential mutations that would be of relevance and make a diagnostic and then sell it back to the country. So the country is a little bit concerned that what may happen is that a company can basically make money off of their data that they’re providing for free. (Interview #1, Lab)*
		3	Access and Who Benefits	Which regions (e.g. LMICs vs. HICs) are actually able to implement WGS technology - including the sequencing itself and the data analysis - and how we avoid situations where these lower-resourced settings are being ‘used’ by other researchers who derive benefit in the form of papers/scientific stature, while the sequences and the resulting data may not ever feed back into meaningful change in TB policy and practice in the study setting. Which regions (e.g. LMICs vs. HICs) are actually able to implement WGS technology - including the sequencing itself and the data analysis - and how we avoid situations where these lower-resourced settings are being ‘used’ by other researchers who derive benefit in the form of papers/scientific stature, while the sequences and the resulting data may not ever feed back into meaningful change in TB policy and practice in the study setting.	*This is all about helping low/middle income countries to get – basically, you know, why should they not be able to reap the benefits of this new technology (Interview #2, Lab).*
		4	Power of Public Health	Actors within the framework of public health can have immense powers which, while working in the public interest, could result in the undermining of various rights and civil liberties.	*Certain things sell, and I think in some ways public health sells, and I just think we need to really make sure that we’re rigorous and when we say something it is of public health benefit - because public health also comes with enormous powers, right - I can look at data, I can look left and let people do things, sometimes you actually, you know basically you have legislation that says you can breach patient confidentiality in the interest of public health - there’s a lot of power associated with public health which means that we have to be absolutely rigorous about that fact that we only use those powers with discretion and we only use it for the purpose for which it was intended. (Interview #10, Governance)*
Emerging concerns in relation to whole genome sequencing, flowing from and mapping back to issues of trust	Bioinformatic challenges	5	Utility	In the context of WGS for TB surveillance, utility is the usefulness or benefit WGS contributes to TB programs both nationally and globally for surveillance, diagnostic and TB care purposes. The utility of WGS within individual TB programs appears to depend on a number of factors including (but not limited to): access to the technology, bioinformatic and human analytic capabilities and engagement with public health workers. The utility of WGS appears to be strongest when it is supported with strong epidemiological data.	*And so, even with whole genome sequencing, like the discriminatory power, it’s just like not that amazing. So, it really, like what it tells you changes so much from disease to disease, it can be really informative in some situations or really not informative in others. (Interview #8, Public Health)*
		6	Analytics	Analytics encompasses many bioinformatic challenges that arise from the use of WGS in TB surveillance and care, including: 1) analytic capabilities of both the technology and the professionals working with it; and 2) standardized processes for standard outputs.	*Whole genome sequencing offers a whole different - It’s a whole different ballgame in terms of resolution power. That said, at this point in time, it’s not fully standardized on how you do the analysis and that’s part of the problem…. it takes you know a while to develop. So right now for whole genome sequencing in its infancy where yes sequencing is now cheap relatively, you can get a pretty good throughput, but you get a ton of information that comes out of it and it’s how you analyze that information and depending on what programs you use to analyze it, you may get different results and therein lies part of the problem. (Interview #9, Lab)*
		7	Capacity	Capacity is the ability of countries to actually implement WGS technology into their local TB programs. The high cost of the machine, the sequencing and/or the analytic software may pose challenges to countries, either alone or in conjunction.	*But it’s then having a pipeline that can actually interrogate that sequence and develop an interpretation of what that sequence means in terms of the resistance in specific genes that we know are associated with resistance. So, you know, we have the platforms to perform whole genome sequencing, but then a pipeline to actually go from the sample through to sequencing to then interrogating that sequence to an interpretation is the bigger challenge. And this is where countries will need a lot more support. (Interview #4, Lab).*
		8	Understanding Science	WGS is a rapidly advancing technology that is often progressing at a rate faster than those working with the technology (either indirectly or directly) are able to adapt. As a result, epidemiologists and other public health workers (i.e.. clinicians and nurses) are often left reliant on the lab staff and others with the niched knowledge of the technology to analyze and interpret the output data. This has the potential to have negative impacts on the overall utility of WGS in public health practice.	*And so, I think as a result probably some risk that the data from whole genome sequencing phylogenetic analysis that we’re providing the many state and local programs in support of their daily practice, is susceptible to misinterpretation just by virtue of the fact that the workforce capacity in terms of training and education has not caught up yet. (Interview #13, Public Health)*
	Data protection and stewardship regarding metadata	9	Metadata	Metadata is the individual patient-level data, including administrative data and health records. Question arise regarding whether or not this data should be linked to sequenced genomic isolates being collected for surveillance purposes and who should have access to this data.	*So what does it mean if we start, you know, that slippery slope, well what happens if we start sharing like well let’s just share gender now, oh let’s share country of origin now. (Interview #7, Public Health).*
			a. Linked Metadata	When metadata databases are linked to the sequenced genomic data produced through WGS, it becomes much more powerful tool for tracking outbreak transmission patterns, at risk case identification and potential diagnostic developments. However, it also raises many questions and concerns regarding privacy and confidentiality of patient level data, as well as data protection responsibilities.	*But we still have to go through our privacy before we can deposit information and one thing that still seems to be a sticking point, not necessarily just with us, I think with other institutions is any associated metadata. So you know I can deposit TB study number one … A sequence, no problem, they know it’s from [province x], but that’s it. But like I said before what’s going to provide the most useful information is all that metadata which will give us context and I think that we’re not there yet in terms of how do we share this information, because really that’s going to be what’s needed. We need worldwide information, so we have an understanding of all the different environments and the different settings where … You know where we see certain drug resistance, mutations* versus *what we see in our population or you know new like strains, clusters, that kind of thing and have that understanding. (Interview #9, Lab)*
			b. Unlinked Metadata	Not linking at least some metadata to the genomic isolates essentially renders the sequenced genomic data produced through WGS useless from a public health perspective as traditional epidemiological activities, such as contact tracing, would still be required to make the data actionable for public health actors.	*So now how do we compare them, so we’re going to need to get a lot of information with respect to you know metadata for example that needs to accompany the whole genome sequence data, like, you know, you need the clinical data, the epi data, you know outcome data and that’s going to shape the knowledge that will come out of whole genome sequencing. (Interview #9, Lab)*
	Consequences and/or implications to data protection responsibilities	10	Stigma	Communities affected by TB tend to already be among marginalized communities. Concerns of further marginalization and stigma arise from the use of WGS for TB surveillance, especially when metadata is linked to the sequenced genomic data. Some of these concerns include: 1) being discriminated against if their identity is discovered through genomic data; 2) public misinterpretation from the media in the reporting of disease outbreaks; and 3) issues of discrimination regarding immigration and care seeking behaviours.	*You know, if we identify sort of questions of transmission – TB, for example – you know, are affecting sort of a particular community or population. What may be the implications of that, how would that potentially perpetuate stigma? I mean, that’s sort of been some of my concerns around how the data’s been used for HIV. And so one of my concerns with the process is that I don’t think, like you say, the ethical considerations are really thought of up front. (Interview #14, Governance)*
		11	Community Engagement	In Canada, the USA and England, TB tends to be disproportionately concentrated within migrant communities, particularly those newly immigrated, and other communities experiencing poverty (i.e.. homeless populations). In Canada, TB is also highly prevalent within Indigenous and First Nations communities. Engagement and transparent communication with these affected communities when reporting out disease outbreaks can help to mitigate some stigma and help ensure that any reports are culturally appropriate and sensitive to the needs of the community.	*And then to communicate with the community, I think we need to have community engagement upfront when we start developing communication pathways. So like now, we need to engage advocacy groups, First Nations groups, representatives of different patient groups, to make sure that their thoughts and concerns are well-represented, and then continue to have them on board when we start communicating these results within communities and between healthcare providers and patients. (Interview #11, Public Health)*
	Challenges assocaited with implementing TB surveillance systems using WGS	12	Implementation/Roll-out	Successful implementation/roll-out of WGS into TB programs for surveillance and care requires that countries have the capacity to implement it, proper analytic systems in place, individuals that understand the science of the technology and the interpretation and finally, buy-in from health care workers that can appropriately communicate it to patients. Additionally, successful implementation of WGS technology depends on: 1) buy-in from state-level and federal administrations; 2) transparency and clear communication strategies for the public and other jurisdictions; and 3) appropriate training systems in place for new professionals working with the technology.	*We don’t get much out of it, it doesn’t seem to make sense, the predictive value is poor, we don’t understand what it’s all about, we don’t use them, we ignore them. We think, oh, all this money is going into typing, and then the end-user totally ignores it, it’s pointless. So it’s really important to explain to them the potential value of using the whole-genome sequence data, and allowing them to feel excited about it, and some ownership around it, and hope that that would mean that they engage with it, and then use it, because otherwise it’s a waste of money. (Interview #16, Lab*).
		13	Jurisdictional Issues	Given greater global connections, TB cases are seldom restricted to one geographic region. Consequently, issues of jurisdiction may occur at the local, state, federal or international level. Issues of jurisdiction may include (but are not limited to): 1) differences in analytic capacity between jurisdiction; 2) ‘ownership’ over the case and responsibility for investigation; 3) communication across jurisdictions and; 4) potential impacts on affected communities including stigma and/or immigration concerns. Consideration of these issues will impact how successfully WGS technologies are implemented into TB surveillance programs.	*But how TB programs are organized, I guess we’ll stick to TB, and actually delivered in each province, that’s their, obviously, their purview and it’s not for anyone, including our government, to comment on. (Interview #6, Governance)*
		14	Communication with non-community stakeholders	Non-community stakeholders includes health care workers (i.e.. clinicians and nurses), laboratory professionals and government officials at state and federal levels. Gaining buy-in and understanding of WGS technology from these stakeholders is vital for successful implementation of this technology. Appropriate messaging of outbreak transmission patterns and WGS technology from TB stakeholders to affected communities should also occur as a result of this.	*This new system is quite visual, so if you present the data in terms of attractive-looking trees, or network diagrams, and explain that to them [TB nurses], then they tend to get it relatively quickly, and enjoy looking at it, and it – I think they get enjoyment out of it, because it makes sense. (Interview #16, Lab)*

The majority of participants identified *trust* as the primary challenge in implementing genomics for TB care and surveillance. More specifically, positive interactions that build or maintain trust between various actors – including but not limited to: persons with TB, healthcare workers, public health administrators, laboratory professionals, diagnostic test developers, and those charged with data stewardship and protection – were discussed as being integral to the relationships required to fully realize the benefits of WGS. For example, one participant highlighted how important it was for the public health community to maintain trust with the public once that trust has been developed:

*“…people have just no problems sending in swabs ….So you can see how comfortable people are getting with it, but I think that’s because there's a certain level of trust, so I think that we in the scientific community and in the medical community have to make sure we don’t betray that trust.” (Interview #9, Lab).*

The trust-based relationships between these various actors shape the ethical challenges as identified by the participants. Some participants also noted, however, that the implementation of WGS must be conducted in a manner that is aware of any historical injustices that might shape the manner in which WGS may be accepted or rejected by local populations. For example, one participant noted that that in the Canadian context, the history of colonialization and higher rates of TB infection in Indigenous communities ought to shape how researchers and clinicians approach said communities:

*“The rates are still higher [in Indigenous persons in Canada] and there's just differences in what all health practitioners need to be aware of in terms of the cultural and historic contexts. A lot of the proliferation of TB that's still residual in older people… [is due to] the legacy of residential schools, right? …. So that's the context. It doesn't change, necessarily, what medications people should be on or who should get followed up in terms of post-contact on a First Nations versus non-First Nations basis. Just the health practitioners are better off if they are aware of that, if they are aware that some older or elderly First Nations people might have been in one of the ‘Indian hospitals’ and might … fear medical practitioners, for very good reasons.” (Interview #17, Governance)*

Participants identified three key ethical challenges that directly stem from issues of trust. First, many participants discussed the *power the enterprise of public health* holds in relation to the general public and persons with TB as an ethical challenge that has the potential to be further heightened as WGS is introduced into national TB programs (NTPs). Participants worried that data derived from WGS could justify public health actions that could infringe on privacy and confidentiality or other civil liberties with insufficient protections. Similarly, a second trust-related challenge several participants identified is a general concern regarding data protection and *data-sharing* responsibilities. Participants discussed, at length, that many organizations, public health institutions, or countries are often reluctant to share even population-level genomic data with other countries or international organizations. One participant suggested that low- and middle-income countries (LMIC) might not fully understand the genomic TB data they have or why they should share it in the first place:

*“…countries simply do not understand the data that they have. They don't understand the value of it, they don't understand the value of sharing it and so they're reacting out of just pure mistrust or a – paranoia is not the right term. I don't know what the right term is, but there's just this – it's sort of this nationalistic sense of we can't share it because it's not the right thing to do” (Interview #2, Lab).*

While this reluctance can be attributed to many factors, including lack of policy or precedent, other participants highlighted that it is most often linked to a historically justified fear of others deriving *profits from surveillance efforts* in the form of new drugs or diagnostics that are then not distributed, or are sold back at a higher price, to those who collected the data.

*“[LMIC are] pretty hesitant [to share] and what has been done in the past is that if they have volunteered to provide data that companies can come in, identify potential mutations that would be of relevance and make a diagnostic and then sell it back to the country. So the country is a little bit concerned that what may happen is that a company can basically make money off of their data that they're providing for free.” (Interview #1, Lab)*

Furthermore, participants indicated that researchers’ interests in advancing their academic careers might also suggest another sense of ‘profit’ that may not be shared equitably with researchers in LMICs.

Finally, participants raised questions regarding which states or jurisdictions are able to access genomics and bioinformatics technology and consequently *implement and benefit* from it. Participants highlighted that trust is particularly relevant in terms of implementing genomics in LMICs without exploiting their residents and their data. Participants stressed that

“…*the most important thing is ensuring the patient gets the best care that they need and deserve here. My point is not to make sure that I get the data from the patient. First and foremost they need to be taken care of and then I’ll figure out how I’ll get the data to try to think more on a population level*” *(Interview #5, Public Health).*

Other participants further claimed that even if the building of WGS tools for TB surveillance and drug susceptibility testing are done in HICs, the benefits of said tools must extend LMICs:

“…*it's absolutely not [about] building something that they will have a disadvantage on… I just want to make sure they have an option to do it simply because otherwise there'll be places that will never see this. They'll never see this technology and I don't think that's right.” (Interview #2, Lab).*

Concerns over the *utility* of WGS in day-to-day clinical practice and surveillance for TB were also highlighted by several participants, particularly by those working in governance or public health roles. Participants described trust and utility as being tied together in that healthcare workers often have to trust the laboratory staff in their sequencing and interpretation of TB genomic data, which currently requires a high level of expertise.

*“I’m not the expert when it comes to the interpretation of the genetic material or the genetic information or what it means, but I suspect that some folks like myself and others in public health sort of take it at face value without necessarily fully understanding all of the limitations of the data.” (Interview #14, Governance).*

As such, the utility of WGS is closely tied to the *analytic* capabilities of a given public health institution – both in the ability to sequence and analyze samples, and the availability of trained staff capable of *understanding the science* associated with the technology and the analytics. Together, the utility, analytics, and ability to understand the science jointly form the *capacity* of any given public health unit to successfully implement genomics safely and effectively for both clinical and public health purposes. It is important to note that when discussing the utility of WGS for TB care and surveillance, participants emphasized that benefits may be imparted at individual, community, or population levels.

While some public health institutions are capable of implementing genome sequencing and analysis, several participants highlighted that any potential analytic capacity is undermined if there is no buy-in from *non-community stakeholders (*i.e.*, those who are not merely members of the general public)*, such as public health administrators and healthcare workers. As many of these stakeholders are acting on behalf of local or national agencies, or directly within the NTPs, participants highlighted that without the support and trust of these stakeholders, integration of WGS into pre-existing health systems would be challenging, if not impossible. More specifically, lack of training in WGS for those already working in the field appears to pose a barrier, as a participant explained:

*“The type of [WGS] data [that] are increasingly used in public health practice. And so, epidemiologists in my generation are required to learn on the job. I say my generation, because I assume graduate schools are now providing infectious disease epidemiologists with the curriculum they need to understand this in their future careers. But for my generation, I think the technology and the science is probably ahead of the training and education.” (Interview #13, Public Health).*

Lack of training in the WGS can limit the understanding of the technology, and thereby limit trust in the technology for this key stakeholder group. Furthermore, several participants highlighted that gaining buy-in from local and regional public health institutions could be achieved by demonstrating the benefits of public health genomics to those working in governance and public health. Participants also noted that such buy-in and trust building at local, regional, and national levels could help mitigate *jurisdictional challenges* raised by WGS-based TB surveillance efforts since outbreaks are rarely confined to one public health region and each region may have their own outbreak investigation protocols.

Moreover, closely related to the utility of WGS for TB care and surveillance, many participants discussed the associated patient-level *metadata* that could be attached to TB genome sequences. Several participants discussed at length that without useful metadata, including clinical, phenotypic, and epidemiological data, genomes alone are of limited utility for tracking TB transmission or deriving new knowledge around genomic correlates of drug resistance. However, when this metadata is linked to a genome, it raises *privacy and confidentiality concerns* and heightens public health units’ data protection responsibilities. Therefore, many participants – particularly those in public health – cautioned that linking metadata requires careful consideration as to the extent of linked data, and taking precautions to avoid further *stigmatizing* already vulnerable populations. As highlighted by this participant, thinking carefully about the optics of the language used in implementing WGS data is critical to minimizing stigma for both individuals and entire communities,

*“Okay, so I think the political and social issues are tied into the ethical issues, but I think one of the major issues is just the optics of it, that we really need to clearly articulate what we’re doing with this [genomic data], because it can infer transmission, certainly, and it can infer person-to-person transmission. So you can actually label a person and call them a “super spreader”, or a “high spreader”. Or you can classify a group of people as super spreaders or high spreaders, which obviously could be stigmatising towards a person, a group, an entire community.” (Interview #11, Public Health).*

Several participants, especially those with governance capacity, suggested that *community engagement* is paramount to building a relationship of trust, thereby enhancing the overall utility of WGS as a tool for NTPs, though the specifics of what processes are entailed by such community engagement was silent even after further probing.

## Discussion

This project is the first to engage stakeholders regarding the ethical challenges of using genomics toward a specific public health end, namely assisting in the TB surveillance and drug susceptibility testing. Others have written about ethical issues related to WGS only conceptually, i.e., without engaging stakeholders, and most often in the context of human genome sequencing for personalized medicine [10, 15], meaning the results may not be extensible to the public health microbiology context. Furthermore, there are currently few government or institutional policy documents that provide practical guidance for handling ethical issues in public health surveillance with regard to genomics technology [16]. As WGS continues to be implemented by NTPs and surveillance programs, the need for practical policy guidance will become paramount.

In the literature, we see that the concept of trust is foundational to the very definition of public health, as defined by the Institute of Health in 1988: “Public health is what we, as a society, do collectively to assure the conditions in which people can be healthy” [17]. Childress and colleagues state that (2002) “‘what we, as a society, do collectively’ suggest(s) the need for cooperative behavior and relationships built on overlapping values and trust” [18]. However, currently the literature only passively alludes to the concept of trust in the context of genomics for both infectious disease care and surveillance purposes, painting a limited picture of what trust means in this context [19]. Specifically, this literature does not provide concrete suggestions through which genomics ought to be implemented for infectious disease surveillance that is cognizant of building trust in public health. While challenges related to engendering trust in new technology integration has been thoroughly examined in relation to biobanking, this discussion is sorely missing from the literature regarding whole genome sequencing [19–22]. In those instances where ‘trust’ has been identified in projects as a key value in the context of emerging data sciences and genomics technologies, the use of trust remains atheoretical [23–25]. In this project on WGS and TB, we, too, use the notion of trust and trustworthiness atheoretically, as a dispositional value and characteristic of persons or groups given the empirical scope of our project. Therefore, further theoretical work on the value of trust is needed, so that it can be more robustly applied in public health and technology implementation. We would argue that we must build on the various discussions of trust in the ethics literature [26–28], and combine it with existing empirical bioethics accounts of trust in data sciences and genomics, to produce normative accounts that are both philosophically sound and practicable.

Based on our interviews, we can draw the following conclusions: first, those persons or groups developing and implementing WGS for public health microbiology need to carefully consider the ecosystem or context in which this technology will be implemented in order to build and maintain trust between key stakeholders, including the public and patients. In large measure, understanding the context includes understanding the power dynamics that exists between actors and the history that may have led to existing power imbalances in the first instance. The interviews in this project suggest that some of those persons working with WGS in TB are aware of the pre-existing contexts in which they aim to build trust, though perhaps not all; even if they do acknowledge power imbalances, it may be that they do not fully account for its current and potential future effects on the implementation of WGS. Moreover, within in the context of an endemic or pandemic infectious disease, it is imperative to come to a shared understanding of what goals public health seeks to achieve via prevention and treatment measure, e.g., TB, to ensure that everyone within a public health unit can direct their efforts accordingly and efficiently. Building and maintaining relationships of trust between public health actors will become essential to building a public health ecosystem in which WGS can thrive.

Part of understanding how to build and maintain trust within a public health ecosystem includes understanding what material, human resources, and general infrastructure currently exist to facilitate the integration of WGS into a given public health system. This includes both the necessary technical expertise, but also the ancillary infrastructure needed for implementation, such as the ability to better communicate within and between public health units and the need to build cross-disciplinary personnel capacity. For example, laboratory technicians with sequencing and bioinformatics expertise need to work with the clinicians and epidemiologists delivering care at the patient and community levels, which can be facilitated by both cross-training and through improved communication between groups, thereby building trust both in the technology and within healthcare teams. Trust in the technology can be fostered through enhanced transparency of the science of genomics for public health workers. Moreover, communication between those working in public health units also means creating the space for dialogue between stakeholders, whereby clinicians can ask questions of laboratory experts, and laboratory experts listen to the needs of clinicians to build more useful genomic and bioinformatics tools.

Furthermore, most participants suggested that we ought to meaningfully engage with members of the public likely to be affected by public health genomics efforts in order to build and maintain their trust. Building trust in WGS and public health is always imperative, but it is particularly timely given the air of mistrust toward scientists [29]. Technological innovations have a history of not succeeding for a number of reasons, one of which is a lack of engagement with communities and the broader public, while not accounting for historical injustices [30, 31]. This public engagement requires, at minimum, two approaches: 1) situating WGS within the broader public dialogue of science, public health, and addressing infectious diseases; and 2) working with patients in specific disease areas. For example, in HIV and TB, civil society has already produced Good Participatory Practice guidelines [32, 33] for involving communities as part of drug development efforts; a similar process can and should be used for the development of genomics-based diagnostics and surveillance tools.

Finally, those using WGS in high-income countries (HICs) need to carefully consider their obligations toward their colleagues and communities in LMICs. There is extensive literature on the need to create mechanisms for data-sharing and ensuring that new technologies are accessible to those working in LMICs [8, 26, 34]. The ethos of granting agencies and foundations, such as the Bill and Melinda Gates Foundation, needs to be heeded: the development of technologies like WGS should not proceed without considering the implications for LMICs and global health [35, 36]. The Relational Sequencing TB Data platform (ReSeqTB) is a global initiative with the goal of providing a “one-stop shop” of global genetic TB isolates, and are thereby providing HICs and LMICs with a mechanism to share TB data globally and enhancing LMIC’s access to genomic technology and data. Although HIC NTPs are using local funds to implement WGS for regional benefits, it is still necessary to consider the global health impacts of these programs. Infectious diseases do not respect borders – indeed it is often said that “TB anywhere is TB everywhere” [37] – thus we must work collaboratively with colleagues in LMICs to better attend to the global scale of infectious diseases. LMICs have experienced a history of exploitation of genetic data and, as a result, are often reluctant to share data within global initiatives [38].

Furthermore, global health governance in the context of public health continues to face significant challenges including, but not limited to: insufficient global coordination, national and organizational self-interest, and a lack of global resources [11]. The complexity and challenges associated with WGS discussed in this paper are likely to only further exacerbate these challenges. In the new era of large-scale data science, including artificial intelligence and machine learning, concerns around what some have termed ‘surveillance capitalism’ may also arise [39], i.e., the profits and tools that are derived from data acquired through surveillance activities that may be cost-prohibitive for countries to acquire for local populations. Therefore, keeping in mind concerns related to surveillance capitalism, it is imperative that HIC and organizations tasked with using WGS for TB surveillance consider these implications for LMICs and work *with* colleagues in LMICs to address global challenges.

### Limitations

Genome sequencing is presently an emerging, rather than an established, tool in the TB laboratory, with its current use and integration into existing health systems limited to three major geographic regions and select international health organizations. Thus, in this exploratory study, the perspectives and experiences shared primarily derive from Canada, the US, and the UK, with a particular emphasis on the Canadian experience. We did find that results did not differ between participants on the basis of geography. However, most participants were based in HIC with low TB incidence rates since that is where WGS technology is currently being used; as such, perspectives from LMIC were only partially or tangentially captured, through the responses of those working for international organizations representing global interests, despite our efforts to identify participants through snowball sampling. We intend to explore the experiences of those working with WGS in LMICs in future research. Finally, due to limited resources and limited patient education on the use of genomics for TB care and surveillance, perspectives of patients with TB were not captured. These perspectives will add valuable insight into ensuring the fair and responsible implementation of genomics into existing health systems, and we intend to explore these perspectives in future research.

## Conclusion

The use of genomics in TB laboratories and TB prevention and care programs is becoming increasingly common, necessitating an inventory of the ethical, legal, and sociopolitical implications associated with public health genomics. Given the complex and technical nature of this new approach to public health microbiology and epidemiology, establishing relationships of trust between those working with genomics technology and those directly impacted by it is imperative in ensuring that this promising new approach is used to its fullest potential.

## Additional files

Additional file 1: Table of interview questions. Semi-structured interview guide. A copy of the semi-structured interview guide broken down by question category, selected questions for each category and relevant potential sub-probes. (DOCX 15 kb)

## Data Availability

The datasets created and/or analyzed during the current study are not publicly available due to confidentiality requirements but can be available from the corresponding author in an anonymized format after consent is obtained from participants.

## Abbreviations

HIV: Human immunodeficiency virus
LMICs: Low and Middle-Income Countries
NTPs: National TB programs
ReSeqTB: Relational Sequencing TB Data platform
TB: Tuberculosis
WGS: Whole genome sequencing
