# Supplementary material for: Trust and the ethical challenges in the use of whole genome sequencing for tuberculosis surveillance: a qualitative study of stakeholder perspectives
Source: BMC Med Ethics. 2019 Jul 4;20:43. doi: 10.1186/s12910-019-0380-z (PMC6610958; doi:10.1186/s12910-019-0380-z)
Supplement: Supplementary file 1 — Table of interview questions. Semi-structured interview guide. A copy of the semi-structured interview guide broken down by question category, selected questions for each category and relevant potential sub-probes. (DOCX 15 kb) [file 12910_2019_380_MOESM1_ESM.docx]

| Question Category | Selected Questions | Sub-probes |
| --- | --- | --- |
| Introduction | Please describe your current role and your history working in whole genome sequencing for infectious disease epidemiology |  |
|  | What are some ethical (social, political) issues related to infectious disease surveillance and outbreak investigation? | Follow up and further probe re: responses to these questions. |
| Risks and Benefits of WGS | What are the risks and benefits associated with the use of whole genome sequencing for TB surveillance? | How do you balance these risks and benefits in your experience working in the field? |
| Global Health Governance | What are the implications of being able to identify TB patients more accurately and more quickly than before using whole genome sequencing? | Do you foresee any specific ‘social, political, public health, or economical’ implications? |
|  | What are the rights and responsibilities of the state vis-à-vis persons with TB and their communities when using human genome sequencing for surveillance purposes? | How do we take precautions to protect privacy and confidentiality of patients with TB? |
|  | What are the rights and responsibilities of agencies vis-à-vis persons with TB in light of the opportunity to detect potential cases faster and with more accuracy using human genome sequencing technology? |  |
|  | What role should international organizations, such as the WHO, play in governing the use of whole genome sequencing in TB outbreak investigations and surveillance? | Who should be the key stakeholders and what responsibilities should they have re: WGS for TB surveillance? |
|  | How do you think the values underpinning the concept of global health governance can inform global health policy regarding the use of whole genome sequencing for TB surveillance moving forward? |  |
